# OXA-CuS@UiO-66-NH_2_ as a drug delivery system for Oxaliplatin to colorectal cancer cells

**DOI:** 10.1007/s10856-021-06574-y

**Published:** 2022-02-28

**Authors:** Marjan Gholami, Azadeh Hekmat, Majid Khazaei, Majid Darroudi

**Affiliations:** 1 Department of Biology, Science and Research Branch, Islamic Azad University, Tehran, Iran; 2 Metabolic Syndrome Research Center, Mashhad University of Medical Sciences, Mashhad, Iran; 3 Nuclear Medicine Research Center, Mashhad University of Medical Sciences, Mashhad, Iran; 4 Department of Medical Biotechnology and Nanotechnology, Faculty of Medicine, Mashhad University of Medical Sciences, Mashhad, Iran

## Abstract

In this work, UiO-66-NH_2_ was used to prepare a new delivery system by incorporating copper sulfide (CuS) into the pores. The CuS nanoparticles (NPs) were prepared to enhance the anticancer effects of Oxaliplatin (OXA) against colorectal cancer. The oxaliplatin was loaded into CuS@UiO-66-NH_2_. To characterize and investigate their cytotoxicity effects, powder X-ray diffraction (PXRD), Fourier transformation infrared spectroscopy (FT-IR), Brunauer–Emmett–Teller (BET) analysis, UV-Visible analysis, inductively coupled plasma mass spectrometry (ICP-MS), and MTT assay were considered to be performed. According to the observations, the cytotoxicity of OXA-CuS@UiO-66-NH_2_ was greater than that of the OXA alone.

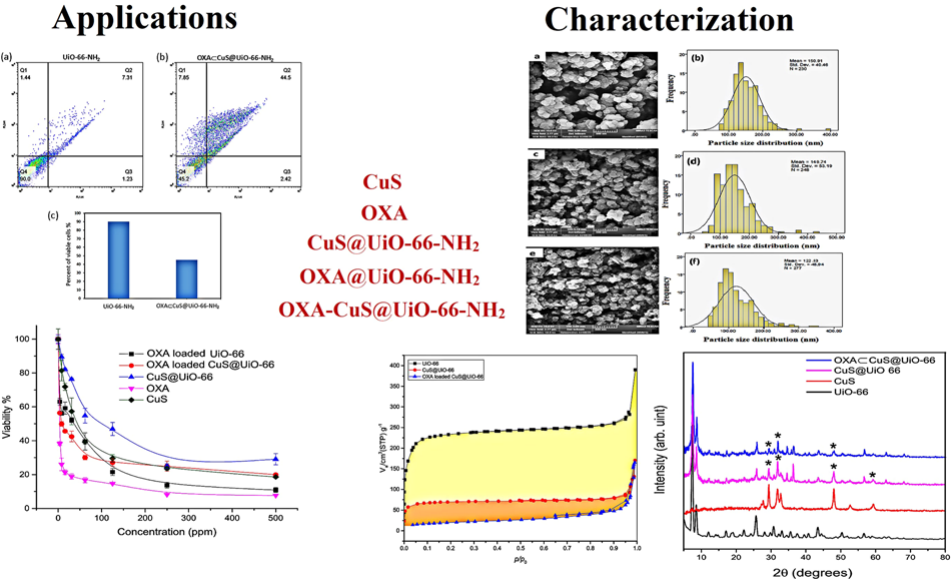

## Introduction

The colorectal cancer (CRC) is one of the major causes of death in the world due to the lack of an effective drug that would cause fewer side effects [1, 2]. However, there have been significant improvements in the field of cancer by the exertion of new techniques in designing drug delivery systems. Novel pharmacological systems have proved to be effective in the targeted and specialized treatment of cancer and provided a new field that involves the usage of porous materials for delivery systems [3–5]. Oxaliplatin is a type of chemotherapy drug that is currently approved for the treatment of colorectal cancers. It is a hydrophilic compound of platinum that, like other platinum derivatives, including cisplatin and carboplatin, inhibits DNA synthesis [6]. The compounds that this drug creates to inhibit DNA synthesis are more toxic than cisplatin and carboplatin. In addition, unlike cisplatin, this drug can inhibit RNA synthesis. However, the in vivo anti-tumor activity of Oxaliplatin has been reported to be low when used alone [7]. This low activity can be attributed to high partitioning to red blood cells and low drug accumulation in tumor tissues after intravenous injection [8]. On the other hand, the clinical efficacy of this drug is limited by its side effects such as neurotoxicity, which are also dose limiters. Side effects of Oxaliplatin and other chemotherapeutic drugs are the result of systemic administration and, as a result, the effect of these drugs on normal cells in addition to cancer cells [9]. Considering the above, it seems necessary to use a new drug delivery system that can reduce the side effects of the drug by entering the tumor site and increasing its anti-tumor activity by preventing it from entering normal tissues [10]. Therefore, one way to overcome these challenges is to use highly targeted chemical drugs using nanocarriers. In this work, the fabrication of CuS@UiO-66-NH_2_ nanomaterials containing Oxaliplatin is considered for inactive targeting. With the remarkable nanoscience development that has been created over the past few decades, porous nanomaterials have been developed [11–13] that can conjointly alleviate various of the aforementioned side-effects when combined via OX into novel drug delivery systems (DDSs). Between the porous substances, metal-organic frameworks (MOFs) are the most flexible in terms of their design flexibility and applicability as novel nanomaterials-based DDSs [13, 14]. The various toxicological shapes which recently introduced nano MOFs displayed negligible toxicity profiles for carboxylate-based nano MOFs [15]. They are sequestered by the liver and spleen, are biodegradable, degraded, and eliminated from the organism with urine or feces [16–18]. Though different MOF structures have been developed, UiO-66 and its functionalized forms (X-UiO-66, NH_2_, NO_2_, etc.) are of important interest as DDSs because of their intrinsic properties [19]. The MOFs-conjugation via another material such as nanoparticles are interesting substitutes that could further increase their potential as the carrier in targeted drug delivery systems [11, 20]. These carriers are widely applied for enhancing the bioavailability, solubility, and controlled release of drugs [21–23]. The nanoporous carriers that are used for the preparation of new pharmaceutics include metal-organic frameworks, which have demonstrated high efficiency in the loading process and caused improvements in drug release [24]. Due to their unique properties such as large and malleable porosity, as well as the presence of modifiable organic groups, it is possible to adjust this framework for specific purposes such as regulating the size of the cavity [24–27]. It is also possible to synthesize metal-based nanoparticles into pores for obtaining new characteristics such as fluorescence to metal-organic frameworks [28–30]. By controlling the factors that affect the release rate of drugs and the amount of drug loading, metal-organic frameworks seem to be very idealistic and exhibit a promising horizon in drug delivery systems [30]. Zirconium-based Metal-organic frameworks, such as UiO-66 and UiO-66-NH_2_, can be considered as a practical alternative for designing drug delivery systems since they contain an adjustable cavity size and high compatibility with physiological systems [30–32]. Some of the advantages of using UiO-66-NH_2_ include obtaining the benefits of biological adaptability and the ability to attract large amounts of drugs, as well as having control over the drug release [32–34]. According to the recent findings, copper sulfide nanoparticles can treat cancer by destroying cancerous tumors and stimulating the immune system against cancerous cells. In addition, the copper sulfide that is sized in a nanometer scale has been observed to display different features than its bulk form and upon being exerted in a controlled manner, they have proved to be safe and antineoplastic. It is also notable that next to directly annihilating the cancer cells, copper compounds cause an enhancement in the immune system to cope with cancer as well [34–40]. In this study, copper sulfide nanoparticles were integrated into the structure of UiO-66-NH_2_ and applied in vitro to deliver Oxaliplatin to the colorectal cancer cells. The performance of this structure was investigated by the means of MTT assay and flow cytometry (FCM).

## Materials and methods

### Chemicals and instruments

All of the chemicals were purchased from Sigma-Aldrich, Inc. and Merck GmbH (Darmstadt, Germany) unless stated otherwise and used without any further purification. Polyethylene glycol was procured from Pars Azma, Iran, while Methanol and ethanol were obtained from Pars alcohol, Iran. The following instruments were used for the experiments of this study: centrifuge (Froilabo Firlabo SW12R), ultrasonic bath (Bandelin Ultrasonic Bath DT 52 H SONOREX DIGITEC 240 W with Heating), X-ray diffractometers equipped with Cu irradiation, λ = 1.5406 Å (X’Pert Pro MPD), field emission scanning electron microscopy (TESCAN MIRA FESEM, Czech Republic), BELSORP MINI X Surface Area and Pore Size Distribution (from Japan), Fourier transform infrared spectrometer (Thermo Nicolet, Avatar 370 FTIR), the inductivity coupled plasma (OPTIMA 7300DV) and dynamic light scattering and zeta potential analyzer (Zetasizer Nano ZS, Malvern Instruments Ltd.).

### Nanoparticle synthesis

#### Synthesis of UiO-66-NH_2_

Initially, 0.52 g of 2-aminoterephthalic acid was dissolved in 40 mL of DMF. Then, 0.50 g of ZrCl_4_ was dissolved in 20 mL of DMF and 4 mL of HCl by the usage of an ultrasonic bath for 20 min. Once the solutions were mixed, the process was continued by performing sonication for another 20 min. The mixture was poured inside a Teflon-lined stainless steel autoclave and heated at 80 °C for 36 h. After being cooled off, the precipitate was separated through centrifugation (3000 rpm/min) to be washed three times with 20 mL of DMF and three times with 20 mL of HCl solutions. The obtained product was set aside at room temperature for two days and activated at 90 °C under vacuum before being used.

#### Incorporation of copper sulfide nanoparticles into the pores CuS@UiO-66-NH_2_

To begin the process, 0.34 g of CuCl_2_ was dissolved in ethanol (15 mL) and ethylene glycol (15 mL) at room temperature. Then, 0.10 g of activated UiO-66-NH_2_ was added to the prepared solution and stirred for an hour at room temperature, which resulted in the formation of a green-blue solution (the first solution). In the following, 30 g of thioacetamide was dissolved in ethanol (10 mL) and ethylene glycol (10 mL) and stirred for 20 min at room temperature (the second solution). The second solution was appended to the first mixture at room temperature and allowed to rest for 1 h to achieve a pale yellow-colored solution. The mixture was heated at a temperature of 150 °C for 24 h in a Teflon-lined stainless steel autoclave. After being cooled off, the obtained precipitate was separated by centrifugation (4000 rpm/min) and washed three times with ethanol (20 mL) after being vortexed for 20 min. As the last step, the product was dried at 60 °C for 12 h and from this point, the resulting powder was labeled and called CuS@UiO-66-NH_2_.

#### Preparation of OXA-CuS@UiO-66-NH_2_ and OXA@UiO-66-NH_2_

As the first step, 1.0 g of CuS@UiO-66-NH_2_ was dispersed in Oxaliplatin solution (5 mL, 5.0 mg/mL) and the suspension was stirred in the dark at room temperature for 48 h. Then, the resultant was centrifuged (4000 rpm/min) and rinsed once with distilled water to be dried later on at room temperature overnight. The obtained product was labeled as OXA-CuS@UiO-66-NH_2_ and meanwhile, a similar procedure was performed to prepare OXA@UiO-66-NH_2_.

#### Cell culture

For this experiment, we procured a CT26 cell line from the Iranian Pasteur Institute. An RPMI medium that contained FBS (10%) and penicillin/streptomycin antibiotics was exerted to culture the cells. The colorectal cancer cells were kept in an incubator chamber at 37 °C with a humidity of 5 and 95% of CO_2_.

#### Growth inhibition studies

The colorectal cancer cells were treated with the prepared Nano drug and its constituents for 24 h; then, 20 µL of MTT solution was added and incubated for 4 h. The MTT was reduced by succinate dehydrogenase during the incubation period, which resulted in the formation of violet formazan crystals. Thereafter, 150 µL of DMSO was appended into each well to solubilize the existing crystal. Finally, the absorption was read and recorded by the application of a plate reader at λ_max_ = 570 nm.

#### Annexin V-FITC/PI staining

CT26 cancer cells were seeded in a 6-well plate (cell density = 7 × 10^4^ cells per well) to be treated with the composed Nano drug for 24 h and be compared to the control group. Then, the cells were suspended in a 200 µL X Annexin V binding buffer. After being centrifuged at 400 × *g* for 5 min, the pellet was separated and suspended in an Annexin V-FITC/PI staining solution. The mixture was incubated at ambient temperature for 15 min and analyzed afterward by the application of FACScalibur flow cytometer and FlowJo software.

## Results

### Powder X-ray diffraction

**Fig. 1:**
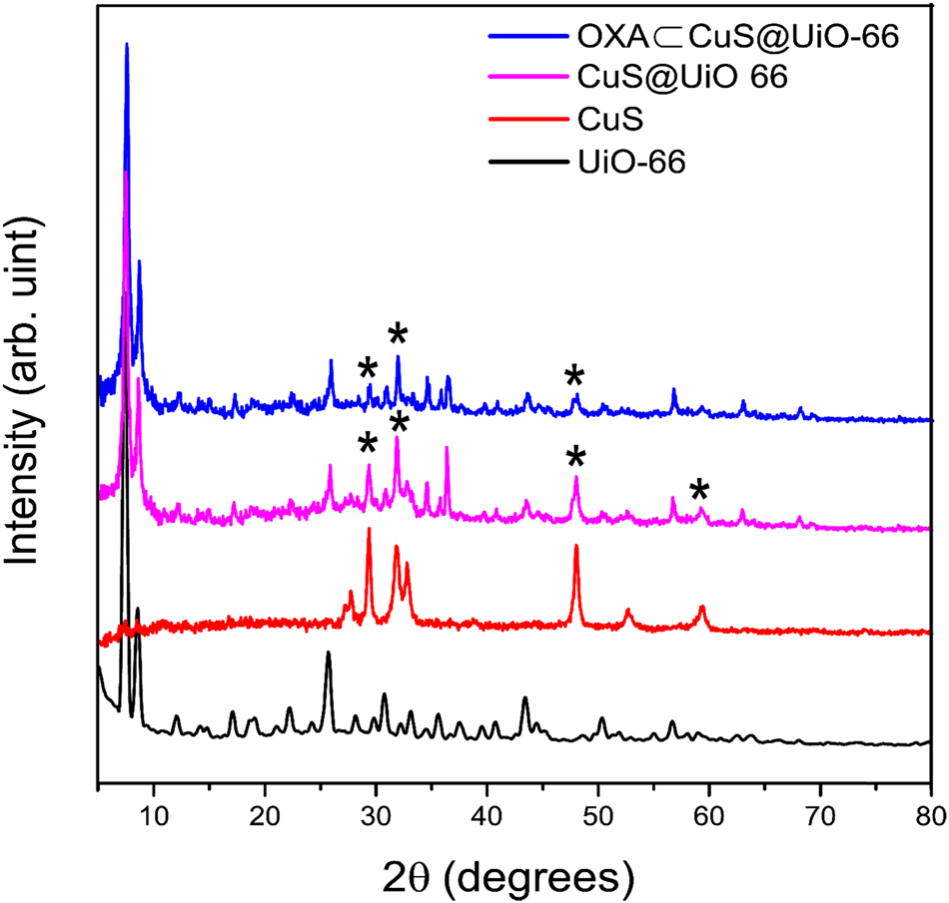
PXRD of UiO-66-NH_2_, CuS, CuS@UiO-66-NH_2_, and OXA-CuS@UiO-66-NH_2_

Diffraction is a phenomenon in which X-rays are scattered by atoms with a crystalline structure in specific spatial directions that reinforce each other and create stronger rays. This phenomenon is the only direct method of determining the crystalline structure and phase of varying materials. The main purpose of a diffraction experiment is to distinguish the angle of each peak and then, specify the distance of the atomic plane. The crystal structure of UiO-66-NH_2_, CuS-UiO-66-NH_2_, OXA-UiO-66-NH_2,_ and OXA-CuS@UiO-66-NH_2_ were compared with the reference patterns of CuS and UiO-66-NH_2_ to monitor the formation of sample and changes that occur in the course of the functionalization process [36]. The UiO-66 structure with the Reference code of RUBTAK02 in Cambridge structural database ^31^ was used to analyze the compatibility of prepared UiO-66-NH_2_ and the structural maintenance of CuS-UiO-66-NH_2_ and OXA-UiO-66-NH_2_ after being functionalized with the calculated pattern (Fig. 1). The characteristic peaks of UiO-66, including 7.3° and 8.5°, remained intact during the CuS synthesis and drug loading which was in correspondence to the calculated ones. Furthermore, the performed assessment on the synthesis of CuS contained a reference number of JCPDS # 01-079-2321 that was related to the hexagonal copper sulfide. According to the gathered data, the 2° values of 27.2° (100), 27.7° (101), 29.4° (102), 31.9° (103), 32.8° (006), 48.0° (110), 52.7° (108), and 59.4° (116) were associated with the synthesis of CuS. The presence of CuS in the CuS-UiO-66-NH_2_ and OXA-CuS@UiO-66-NH_2_ was proved by observing certain peaks at 29.4°, 32.8°, 48.0°, and 59.4°. The crystallite sizes of UiO-66-NH_2_, CuS-UiO-66-NH_2,_ and OXA-CuS@UiO-66-NH_2_ were calculated by the Scherrer’s equation and reported to be 17.9, 22.3, and 21.7 nm, respectively.

### Fourier transform infrared spectroscopy

**Fig. 2:**
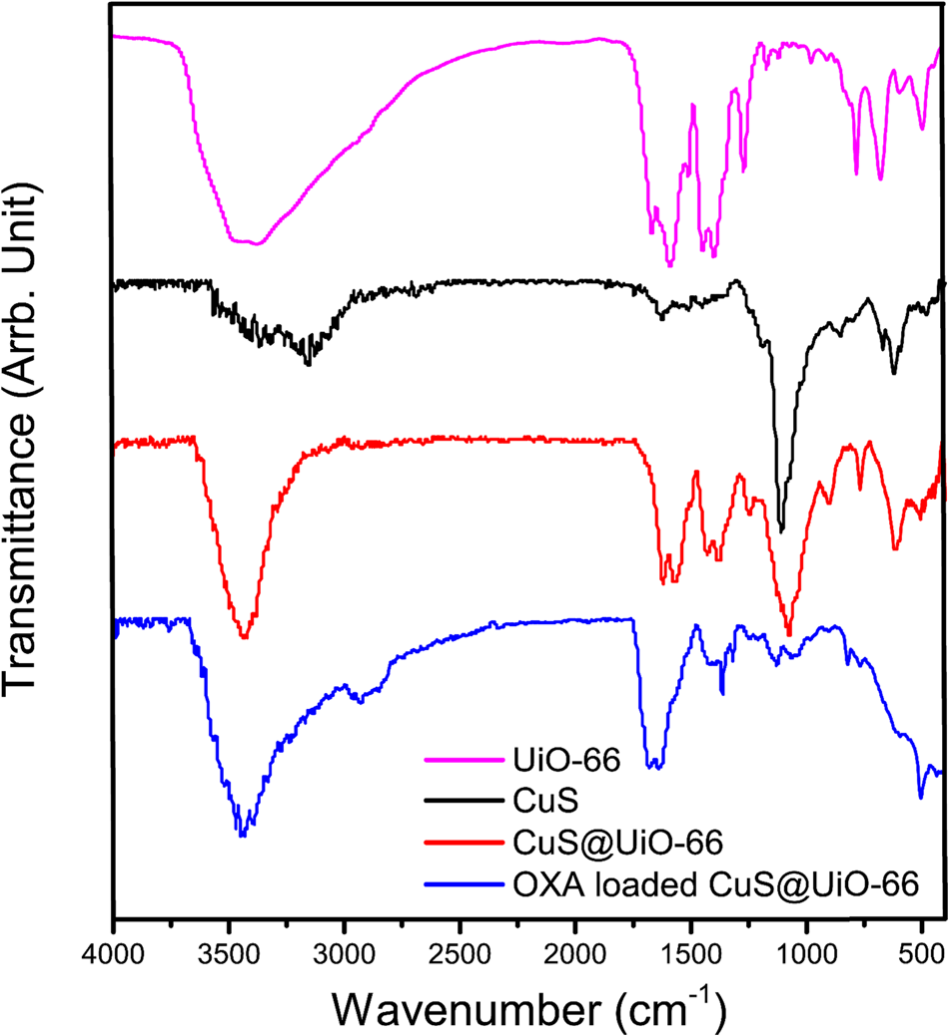
FTIR Spectra of CuS, UiO-66-NH_2_, Cus@-UiO-66-NH_2_, and OXA-CuS@UiO-66-NH_2_

Fourier transform infrared spectroscopy (FTIR) is a well-known technique applied for the identification of different materials (Fig. 2). The most important application of FTIR is the performance of a qualitative analysis that includes identifying the functional groups and determining the structure of organic, inorganic, or hybrid species [21]. The FTIR spectrum of UiO-66-NH_2_ was consistent with the results that had been reported [39, 41] and used for monitoring the changes that occurred during the synthesizing process and encapsulation. The existence of OXA in UiO-66-NH_2_ has been confirmed since the OXA loaded UiO-66-NH_2_ had exhibited aliphatic C-H stretchings at 2850–2990 cm^−1^ after OXA encapsulation. The successful synthesis of CuS has been also approved by the appearance of peaks at 621 cm^−1,^ which had been related to CuS vibrations that appeared as a shoulder on the OXA-CuS@UiO-66-NH_2_ curve at 597 cm^−1^.

### Brunauer-Emmett-Teller surface area analysis

**Fig. 3:**
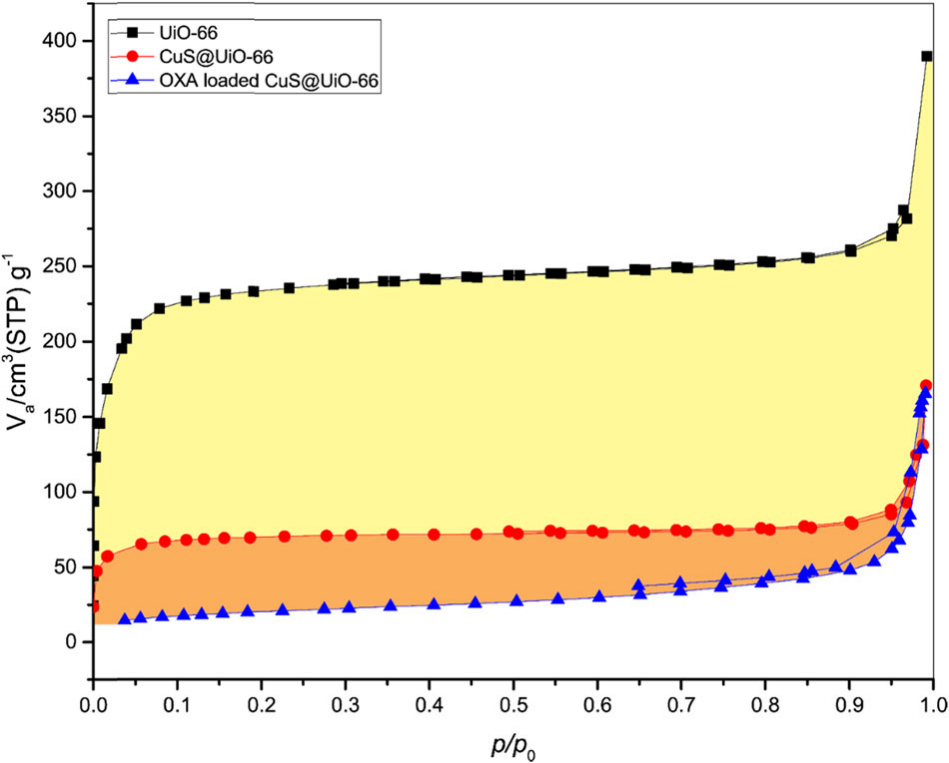
The BET analysis of samples

Brunauer-Emmett-Teller **(**BET) analysis is one of the methods exerted for measuring and evaluating surface porosity since the accurate measurement of surface area and cavities in many applications, such as catalysts, Nano adsorbents, compounds and additives, pharmaceutical, and food industries as well as nanostructures including metal nanoparticles, nanotubes, nanofibers, etc. is considered of great importance. Among the different methods that are available for determining the porosity, the BET method is known to be based on gas absorption. Herein, the results of this particular analysis exhibited the differences in surface area and porosity before and after functionalization (Fig. 3 and Table 1). Accordingly, it can be perceived that the surface area has faced a significant decrease after functionalization. The most visible changes have occurred after the incorporation of CuS into the UiO-66-NH_2_ pores. A decrease in pore size and total pore volume after CuS incorporation is indicative of CuS penetration into the pores while the internal surface area was involved as well. However, there have not been signs of alterations in the pore volume and pore size after the process of drug loading. The attachment of OXA could be highlighted as the most plausible conclusion of this work, which was achieved by exerting the hydrogen bond that exists on the surface of OXA-CuS@UiO-66-NH_2_, leading to a significant decrease in the surface area after the loading since the windows had been blocked by OXA. It is also notable that the presence of CuS can prevent drug penetration into the pores. We have detected a lower loading percentage in comparison with that of the UiO-66-NH_2_, which could be due to the inability of CuS@UiO-66-NH_2_ in utilizing its internal surface area after having CuS synthesized into the pores.

**Table 1:** Surface area, pore size, and total pore of the UiO-66-NH_2_, CuS@UiO-66-NH_2_, and OXA-CuS@UiO-66-NH_2_

Sample	Surface area (m^2^/g)	Pore size (Å)	Total pore volume (p/p_0_ = 0.990)
UiO-66-NH_2_	845.9	27.8	0.5874
CuS@UiO-66-NH_2_	357.7	18.5	0.2406
OXA-CuS@UiO-66-NH_2_	69.0	18.5	0.2527

### Field emission scanning electron microscopy

**Fig. 4:**
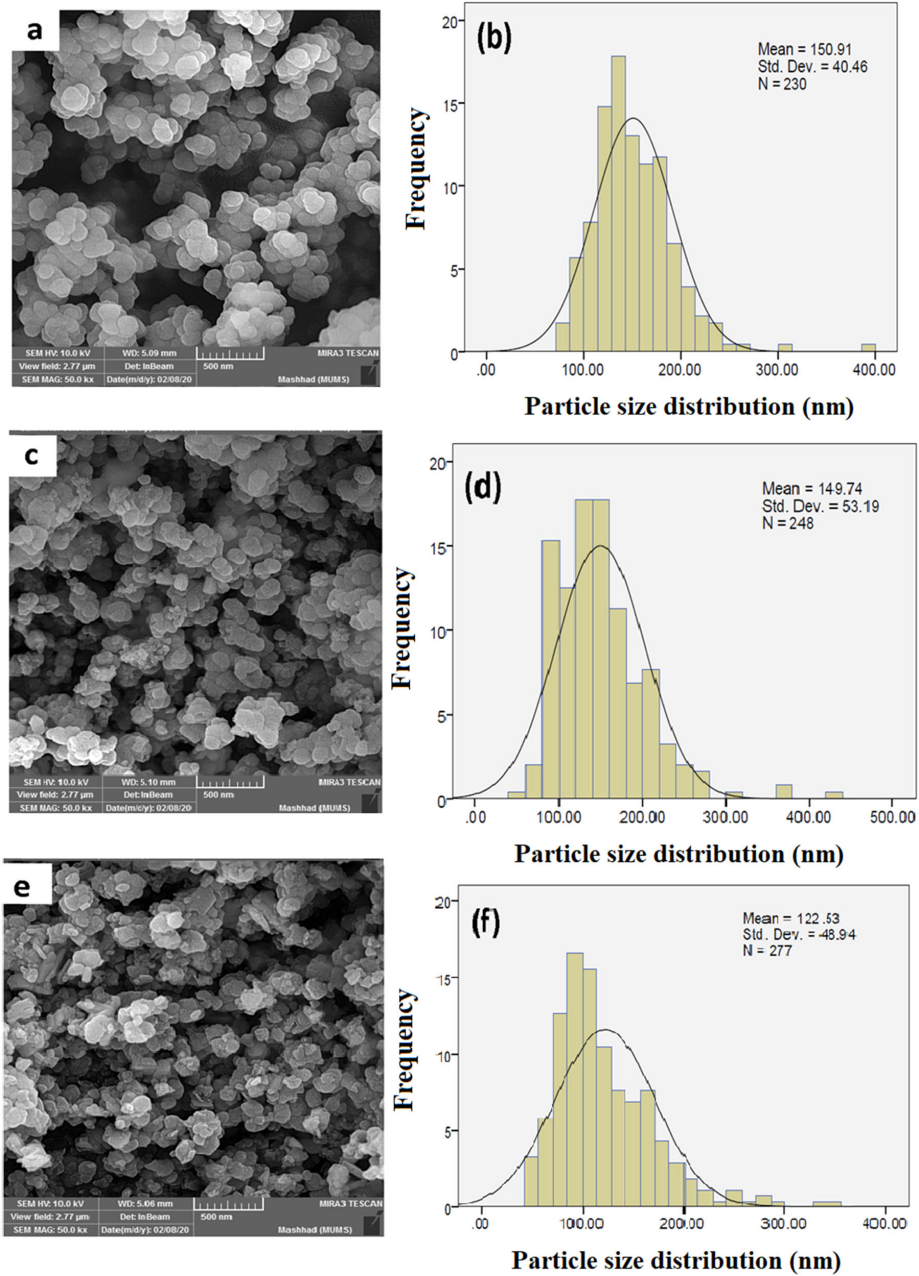
FESEM images and size distributions of the UiO-66-NH_2_, CuS@UiO-66-NH_2_, and OXA-CuS@UiO-66-NH_2_

Field emission scanning electron microscopy (FESEM) analysis is one of the most widely used methods for studying the surface and morphology of materials. The spherical morphology, which can be observed in the FESEM images, was not altered even after functionalization (Fig. 4). The mean particle size distributions of UiO-66-NH_2_, CuS@UiO-66-NH_2_, and OXA-CuS@UiO-66-NH_2_ were measured to be 150.9 ± 40.46, 149.7 ± 53.19, and 122.5 ± 48.94 nm, respectively; however, the particles appeared in lower sizes after the process of drug loading. Mechanical stirring resulted in decreasing the grain sizes obtained from the solid phase surface analysis. The aggregation of UiO-66-NH_2_ and other nanoparticles can be indicated by observing the comparison between the crystallite size and the grain size obtained from FESEM images.

### Dynamic light scattering (DLS)

**Fig. 5:**
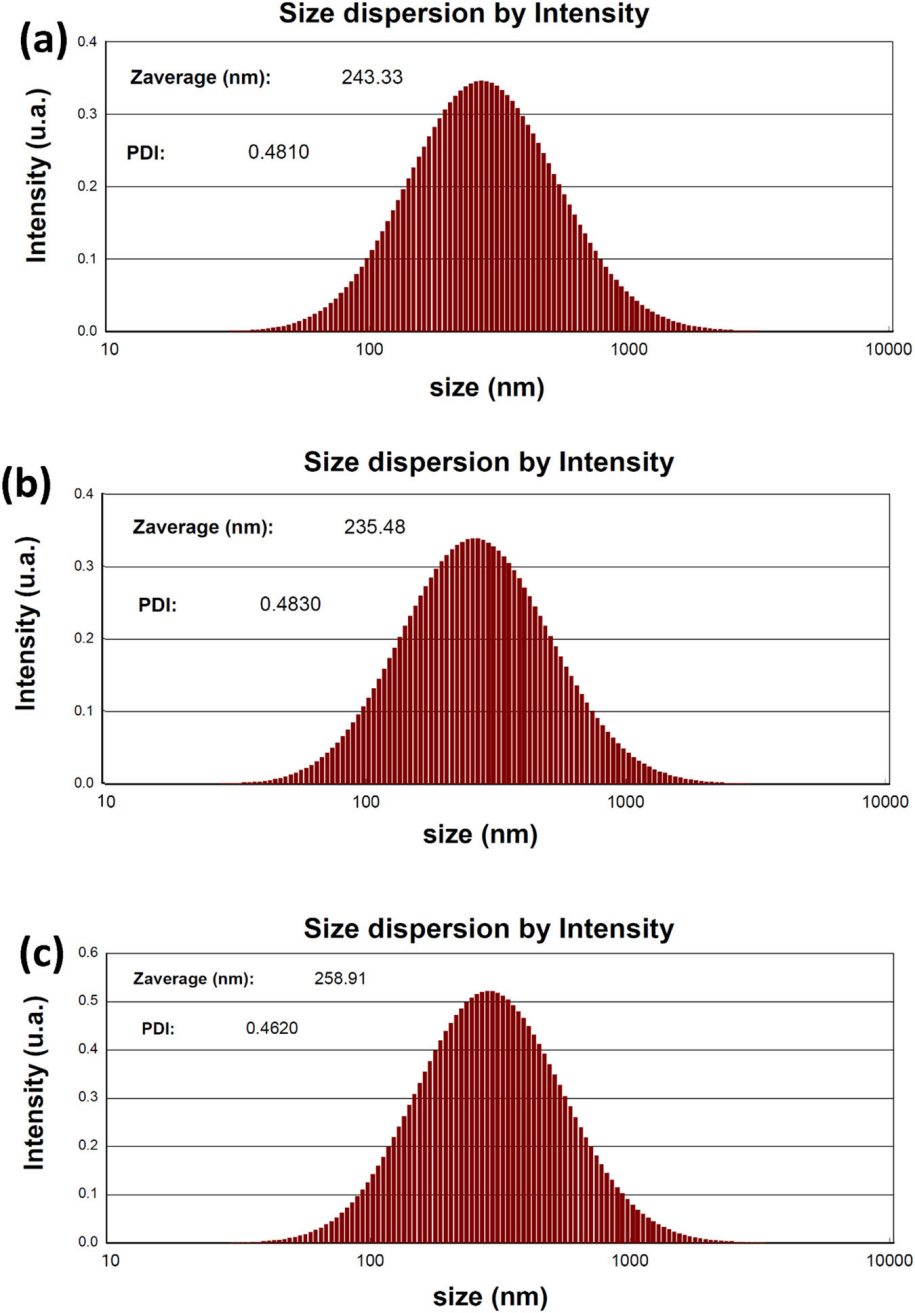
DLS distribution in water and growth medium of UiO-66-NH_2_, CuS@UiO-66-NH_2_, and OXA-CuS@UiO-66-NH_2_

After measuring the results of DLS analyses, we discovered signs of particle aggregation throughout the solution (Fig. 5). In addition, we observed higher amounts of aggregation in sizes that were over 10 times larger than the crystallite sizes, while the dynamic particle sizes were larger than the solid form as well. The probable reasons for this phenomenon include the interaction of solvent molecules with the surface of nanoparticles and the presence of amine functionals on the MOF. Therefore, it could be assumed that hydrogen bonds stand as the most important interaction in the dynamic sizes of prepared nanoparticles. According to Fig. 5, there was no evident increase in the particle sizes after the modification process, and consequently, any conclusion associated with a tendency to increase or decrease in sizes after modifications is denied.

### OXA-UiO-66-NH_2_ and OXA-CuS@UiO-66-NH_2_ MTT assay

**Fig. 6:**
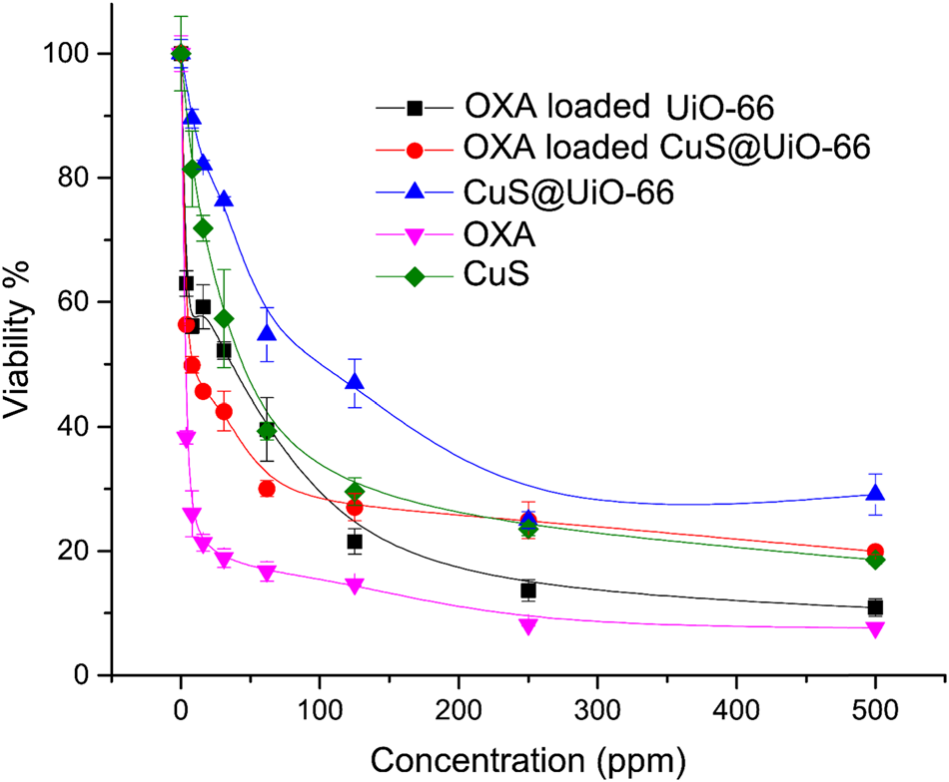
MTT assay of CuS, OXA, CuS@UiO-66, OXA@UiO-66-NH_2_, and OXA-CuS@UiO-66-NH_2_

To analyze the results of survival measurement tests such as the MTT assay, we exerted Elisa as a plate reader device (Fig. 6). In this method, the mean absorption of three wells for each dosage is evaluated. Furthermore, the mean absorption of each dosage minus the amount of blank was also used for further calculations (generally done by Elisa reader). To complete this measuring process, the mean absorption of each dosage was divided by the mean absorption of control wells to be multiplied by 100. The calculated numbers represent a percentage of survival rate or Viability. The lack of any significant cytotoxicity from UiO-66-NH_2_ was indicated by the results of MTT. The IC_50_ of OXA-CuS@UiO-66-NH_2_ was reported to be 7.97 ppm, which is lower than that of the OXA-UiO-66-NH_2_ (37.58 ppm), suggesting that the presence of CuS led to a higher percentage of cellular death. After the loading of OXA obtained by ICP (10.8 and 26.1 %), the concentrations of OXA based IC_50_ were 0.8 ppm and 9.8 ppm for the cases of OXA-CuS@UiO-66-NH_2_ and OXA-UiO-66-NH_2_, respectively; as it can be observed, OXA-CuS@UiO-66-NH_2_ has been more effective than OXA (IC_50_ ± 1.05 ppm). Regarding how the IC_50_ of CuS and CuS@UiO-66-NH_2_ were 40.7 and 85.1 ppm, respectively, the cytotoxicity of CuS against colorectal cancer cells is quite evident, which also displayed signs of synergism throughout the treatment process at lower doses.

### Flow cytometry

**Fig. 7:**
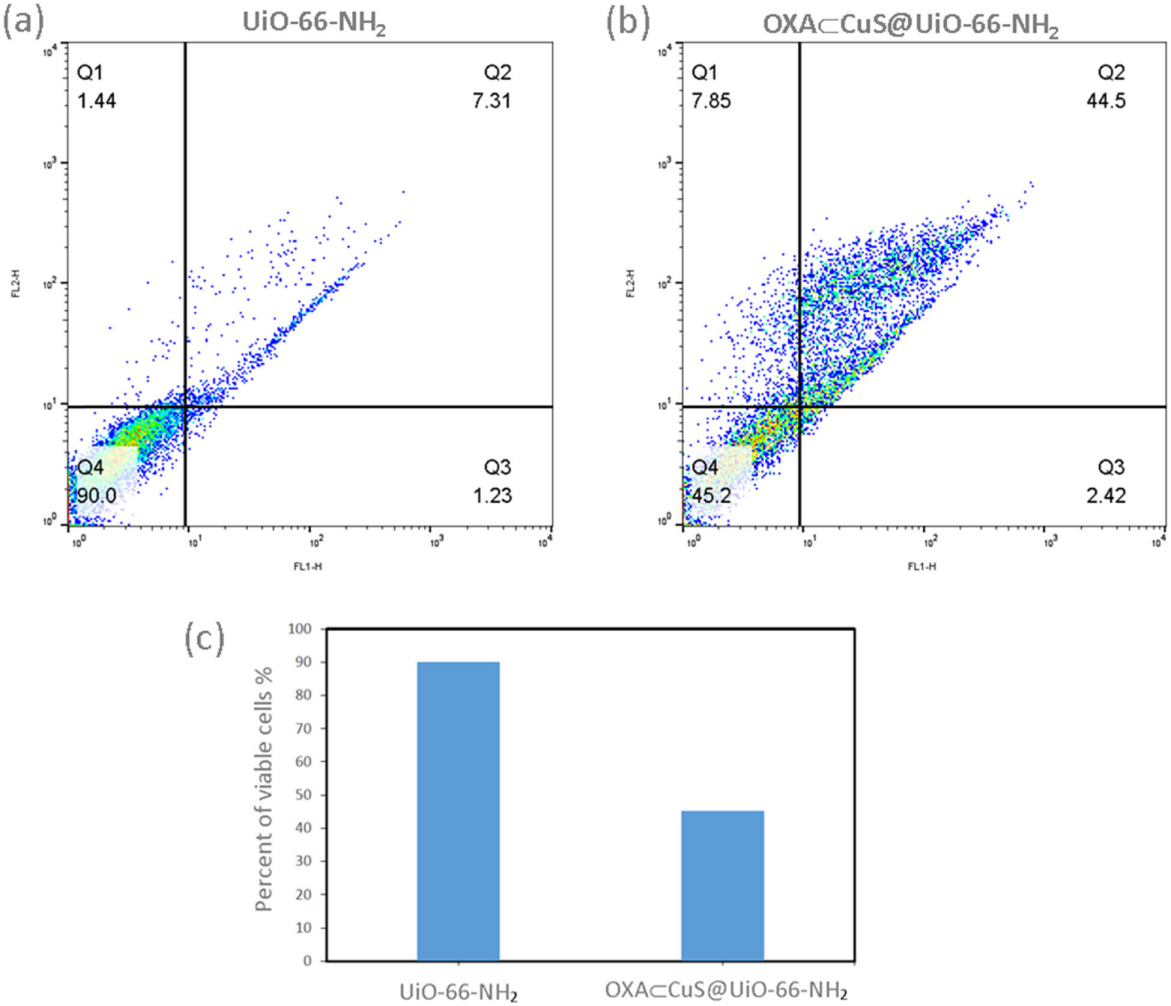
Flow cytometry charts of UiO-66-NH_2_ and OXA-CuS@UiO-66-NH_2_ (**a** and **b**) and statistics (**c**)

The effects of OXA-CuS@UiO-66-NH_2_ on the apoptosis process of the CT26 cell line were investigated by the means of flow cytometry (Fig. 7). After treating the cell for 48 h with a dosage of 100 nM, the amount of apoptosis was calculated through the application of Flow Jo software. According to the results, OXA-CuS@UiO-66-NH_2_ is capable of stimulating the apoptosis process of the CT26 cell line [42].

## Discussion

To design drug delivery systems using MOFs, the MOF and guest interactions are needed to be optimized to control spontaneous drug release [14, 17, 23, 43, 44]. The MOF nanocomposites appear to be effective for this purpose. Using metal oxide/MOF nanocomposites such as incorporated magnetic NPs [45] can be used to facilitate drug release by a stimulant. For example, nimesulide-loaded Fe_3_O_4_@HKUST-1 has been used in the treatment of pancreatic cancer, where the release has lasted over 11 days [46]. γ-Fe_2_O_3_@MIL-53(Al) was also investigated for controlled ibuprofen release which was over 7 days [47]. The other nanomaterials were gold nanorods that were incorporated into [Al(OH)(1,4-ndc)]_n_, which was also controlled anthracene release [48]. The hybrid nanomaterials including metal oxide/MOFs nanocomposites appear to be promising and effective in inducing drug release by a stimulus. However, Few reports presented them for drug delivery and further investigation is needed to fully understand their potential. Copper sulfide (CuS) is a theranostic agent [49] that can be conjugated with drugs and used for imaging of the tumor microenvironment which can complete the lackings of the MOFs for tracking and monitoring purposes. CuS NPs can be used to design drug delivery systems based on metal oxide/MOF nanocomposites for theranostic purposes, which could also lead to a better release profile. Herein, the CuS incorporated UiO-66 was used for the first time to investigate the characteristic of the OXA-CuS@UiO-66-NH_2_ in the killing of the CT-26 cells. PXRD analyses have shown the successful synthesis of the MOF and also CuS nanoparticles. To make sure the CuS NPs were formed into the pores of the MOF, the bet analyses were taken. It was revealed that surface area and the pore size were decreased after the synthesis of CuS NPs. It can be deduced the metal oxide occupied the pore and even closed some windows. After drug loading, FTIR analyses confirmed the presence of C-H stretching of the OXA ligands. PXRD also confirmed that the structure was remained intact after drug loading. Considering the crystallite, FESEM, and hydrodynamic sizes, it appears aggregation is much more intense in the aqueous media. Despite all these obstacles, the samples were meticulously prepared and the powders were thoroughly suspended in the solution for biological tests. The cytotoxicity assay has displayed the effectiveness of the prepared drug delivery system in the killing of the cancer cells concerning OXA IC_50_. It appears the presence of CuS into the pores could work synergistically with the loaded drug. The flow cytometry also confirmed the aforementioned results.

## Conclusion

In conformity to the results of analyzing the synthesized OXA-CuS@UiO-66-NH_2_ by the BET method, the formation of CuS within the pores was successfully achieved, however, it led to the lack of using the internal surface for OXA loading. Furthermore, the presence of crystallites aggregation caused an increase in the solid phase and dynamic sizes. In addition, the existence of hydrogen bonds resulted in intensifying the dynamic sizes and caused the 10 percent adsorption of OXA, which was indicated through the ICP analysis. It was also notable that apparently, the higher loading of OXA in UiO-66-NH_2_ was due to the internal surface area. Despite all of these facts, it is interesting that CuS was able to act as a cytotoxic agent with more efficacy as a result of being exerted in combination with OXA. This work suggests that the prepared Nano capsule could function more efficiently if the fluorescence of nanoparticles or their photothermal properties were also investigated.
